# Population Policy: Abortion and Modern Contraception Are Substitutes

**DOI:** 10.1007/s13524-016-0492-8

**Published:** 2016-07-06

**Authors:** Grant Miller, Christine Valente

**Affiliations:** 1 School of Medicine, Freeman Spogli Institute for International Studies, and Stanford Institute for Economic Policy Research, Stanford University, 117 Encina Commons, Stanford, CA 94305-6019 USA; 2 National Bureau of Economic Research, Cambridge, MA USA; 3 Department of Economics, University of Bristol, 8 Woodland Road, BS8 1TN, Bristol, United Kingdom; 4 Institute for the Study of Labor, Bonn, Germany

**Keywords:** Abortion, Contraception, Nepal

## Abstract

A longstanding debate exists in population policy about the relationship between modern contraception and abortion. Although theory predicts that they should be substitutes, the empirical evidence is difficult to interpret. What is required is a large-scale intervention that alters the supply (or full price) of one or the other and, importantly, that does so in isolation (reproductive health programs often bundle primary health care and family planning—and in some instances, abortion services). In this article, we study Nepal’s 2004 legalization of abortion provision and subsequent expansion of abortion services, an unusual and rapidly implemented policy meeting these requirements. Using four waves of rich individual-level data representative of fertile-age Nepalese women, we find robust evidence of substitution between modern contraception and abortion. This finding has important implications for public policy and foreign aid, suggesting that an effective strategy for reducing expensive and potentially unsafe abortions may be to expand the supply of modern contraceptives.

**Electronic supplementary material:**

The online version of this article (doi:10.1007/s13524-016-0492-8) contains supplementary material, which is available to authorized users.

## Introduction

A longstanding debate exists in reproductive health circles about the relationship between modern contraception and abortion use. Over several decades, population scholars have documented concomitant increases in both contraceptive prevalence and abortion rates around the world in settings as diverse as Cuba, South Korea, Bangladesh, Singapore, Netherlands, Denmark, and the United States (Marston and Cleland 2003; Noble and Potts 1996; Rahman et al. 2001). This phenomenon is commonly attributed to rapid reductions in desired fertility, which in turn increase demand for all methods of birth control (Marston and Cleland 2003).

However, theory predicts that with demand for birth control held constant (and absent absolute moral or religious constraints), women (couples)^1^ will use modern contraceptives and abortion interchangeably: that is, they are substitutes (Bongaarts and Westoff 2000; Kane and Staiger 1996; Marston and Cleland 2003; Rahman et al. 2001; Westoff 2000; Westoff et al. 1981).^2^ A relative increase in the affordability, availability, or acceptability of one should lead women who wish to regulate their fertility to substitute away from the other.^3^ Since the mid-1990s (as declining fertility rates have plateaued), global contraceptive prevalence has continued to rise, while abortion rates have declined—a relationship consistent with substitution.

Debate about the relationship between contraception and abortion has fundamental implications for public policy and foreign aid. Importantly, if modern contraceptives and abortions are substitutes, then an effective strategy for reducing expensive and potentially life-threatening abortions may be to boost the supply^4^ of modern contraceptives. Two recent analyses of the United States’ Mexico City Policy (MCP) suggested that by reducing funding for family planning programs, the MCP may have actually reduced the availability of modern contraceptives relative to abortion and thus increased abortion rates (Bendavid et al. 2011; Jones 2015).^5^

Understanding the tradeoff between contraception and abortion would also shed light on ways to prevent maternal deaths. Research on the determinants of maternal mortality worldwide suggests that unsafe abortion plays a quantitatively important role. In Latin American and Caribbean countries, a systematic review found that unsafe abortion accounts for roughly 50 % more maternal deaths than better-known complications, such as sepsis (Khan et al. 2006). The World Health Organization (WHO) estimated that 13 % of maternal deaths worldwide are linked to unsafe abortion (WHO 2010). Given concerns about underreporting, evidence is also suspected to underestimate mortality from unsafe abortion (Gerdts et al. 2013).

What is needed to establish whether the use of modern contraceptives and abortions are complements or substitutes is a large-scale intervention that alters the supply of one or the other and, importantly, that does so in isolation. To date, finding such cases has been challenging because real-world reproductive health programs generally deliver a bundle of services, making it difficult to disentangle the effect of supply of modern contraceptives or abortion from other program components. As a case in point, the well-known Matlab Family Planning Experiment bundled the provision of modern contraceptives with the provision of both abortion services (menstrual regulation)^6^ and child health services, making it difficult to isolate the effect of contraceptive supply (Miller and Babiarz 2016; Rahman et al. 2001).

This article studies an unusual policy change well suited to assessing the relationship between the use of modern contraceptives and abortion. Starting in March 2004, Nepal legalized the provision of abortion by selected existing health service providers. In addition to its scale, what distinguishes this policy is that in doing so, Nepal did not expand the supply of modern contraceptives, bundle the legalization of abortion with changes in the provision of any other type of service, or expand the health care workforce. We use unusually rich individual-level data representative of fertile-age Nepalese women collected in four waves both before and after the legalization of abortion to estimate how the use of modern contraceptives (and other reproductive behaviors) responded to this policy.

We find that the addition of a legal abortion center in one’s district is associated with a 2.6 % decrease in the odds of using any contraceptive (odds ratio (OR) = 0.974; 95 % CI = 0.961, 0.987), implying that a move from 0 to the mean number of centers post-legalization was associated with a reduction in contraceptive prevalence of 2 percentage points—6 % of the pre-legalization prevalence rate. Decomposing this effect among traditional contraceptive methods (such as withdrawal and the rhythm method), female sterilization, and reversible modern methods, we find that the decrease occurs principally among reversible modern methods.

## Background

### Global and Regional Trends

Globally, contraceptive use and abortion rates have been inversely related over the past several decades. Contraceptive prevalence has increased steadily over the past 20 years, rising from 54.8 % to 63.3 % between 1990 and 2010 (Alkema et al. 2013). Simultaneously, abortion rates have declined steadily, falling from 35 to 28 abortions per 1,000 women on average worldwide between 1995 and 2008 (Sedgh et al. 2012). These global trends are, of course, consistent with substitution of modern contraception for abortion, but a number of potentially important confounding factors have also been at work over time (changes in desired fertility, for example).

The inverse relationship between abortion and contraception is particularly evident in formerly socialist Eastern European countries. Under communism, abortion was a major (if not principal) method of birth control across much of Eastern Europe and Central Asia (Frejka 1983).^7^ After the collapse of communism, abortion rates declined steeply with the diffusion of modern contraceptives during the 1990s (Pop-Eleches 2010; Westoff 2000; Westoff et al. 1998), also suggesting that contraception and abortion may have been used interchangeably.

On the other hand, concomitant increases in both contraceptive prevalence and abortion rates have been observed in a variety of countries further back in time, including Cuba, South Korea, Bangladesh, Singapore, the Netherlands, Denmark, and the United States (Marston and Cleland 2003; Noble and Potts 1996; Rahman et al. 2001). Bongaarts and Westoff (2000) and Marston and Cleland (2003) suggested that these simultaneous increases may occur during transitions to lower fertility if the supply of modern contraceptives fails to keep pace with the reduction in desired fertility. Then, as desired fertility plateaus, substitution between modern contraceptives and abortion should become more evident (Marston and Cleland 2003). This is consistent with global trends since the mid-1990s as the worldwide decline in fertility decelerated (World Development Indicators 2014).

### Previous Estimates of Substitution Between Abortion and Contraception

Many studies of the relationship between contraception and abortion in developing countries are limited to informal analyses of their co-movement. Only a handful of studies have attempted to estimate the causal relationship between the two. Two recent studies investigated changes in abortion and contraceptive use induced by MCP. Bendavid et al. (2011) compared changes in abortion and contraceptive use over time in countries highly exposed to MCP relative to less-exposed countries. The authors find that more exposed countries experienced slower increases in contraceptive prevalence and higher increases in abortion after the reenactment of the MCP, suggesting that reduced contraceptive supply may have increased the incidence of abortion. Jones (2015) compared abortion rates among women in Ghana during periods when the MCP was both enforced and not enforced. She found that rural women were more likely to have an abortion during periods of enforcement, which she linked to the increased number of unwanted pregnancies following the reduction in contraceptive supply under the policy.

Rahman et al. (2001) analyzed changes in abortion linked to the Matlab Family Planning Experiment intervention, finding that abortion rates fell in treatment villages relative to control villages between 1979 and 1998 (despite increasing secular trends in both contraceptive use and abortion). However, the experimental treatment bundled menstrual regulation services with the provision of modern contraceptives between 1977 and 1983 (donors then stopped supporting this component of the program). Most of the relative decline in abortion in treatment areas occurred around 1983 and thus is plausibly due to the end of abortion services. Antenatal and child health services were also bundled with the provision of modern contraceptives beginning in 1978 (Phillips et al. 1984), making it difficult to disentangle the independent contribution of contraceptive supply from improvements in child survival.

Evidence from wealthy countries is also thin. Ananat and Hungerman (2012) found that the availability of oral contraceptives starting at age 16 is associated with a reduction in the probability of reporting having had an abortion between ages 16 and 19. Glasier et al. (2004) found no change in abortion rates in Scottish communities following free distribution of advance emergency contraception to women ages 16–29. Finally, Durrance (2013) analyzed the diffusion of emergency contraception through pharmacies in the state of Washington, finding no change in the abortion rate.

## The Nepalese Natural Experiment

### The Legalization of Abortion in Nepal

Prior to 2002, Nepalese women who terminated their pregnancies faced imprisonment for infanticide.^8^ On September 27, 2002, the King of Nepal signed a bill legalizing abortion prior to the 12th week of pregnancy, prior to the 18th week in cases of rape or incest, and at any gestational age with appropriate medical advice (to protect the health of the mother or in cases of severe birth defects, for example) (MOHP et al. 2006). When this law was enacted, however, Nepalese reproductive health providers were neither permitted nor adequately trained to begin offering safe abortion services. Consequently, there was very little increase in abortion, if any, following this law in 2002 (Valente 2014).

Nepal’s first legal abortion services were offered in March 2004, and the number of health centers registered to provide them grew rapidly over time, rising to 141 in June 2006 and 291 by February 2010. To place this expansion into context, the number of registered abortion providers grew from none to nearly twice as many providers per capita as in the United States by 2010 in a period of just six years.^9^ This large-scale policy change has been hailed by advocates as a success, and according to observers, “Nepal’s experience making high-quality abortion care widely accessible in a short period of time offers important lessons for other countries seeking to reduce maternal mortality and morbidity from unsafe abortion” (Samandari et al. 2012:1).

Under the policy, senior gynecologists from central and regional hospitals as well as from some non-governmental organizations (NGOs) and private clinics were trained to become both the first legal abortion providers as well as safe abortion trainers themselves. With the aim of rapid national scale-up, training then cascaded from regional and zonal hospitals to public district hospitals (Samandari et al. 2012). The private sector (primarily Marie Stopes International and the Family Planning Association of Nepal) also “fill[s] an important niche in urban areas” (Samandari et al. 2012:4) and is less prevalent in rural areas, which were home to 83 % of the Nepalese population according to the 2011 population census. As a result, more populous districts, districts in the more accessible regions of the country, and urban areas were more likely to have legal abortion services in early years. In addition, Nepal experienced a Maoist insurgency in 1996, which led to a 10-year conflict of low to medium intensity that peaked in 2002. Conflict areas between 2004 and 2006 may have also experienced slower, less intense increases in the supply of legal abortion. If areas in which abortion supply grew more slowly had preexisting trend differences in contraceptive use, this could bias our estimates. In the section on Assessment of Robustness and Extensions, we show that our results are robust to allowing for more populous districts, districts in more accessible regions of the country, and urban areas to experience differential time trends in contraception as well as to controlling for conflict intensity.

Although illegal abortions have always been available to some degree, legalization greatly reduced the effective (quality-adjusted) full price. The cost of a legal abortion ranges from 800 Rs to 2,000 Rs ($11.33 USD to $28.33 USD) (MOHP and CREHPA 2007) relative to mean annual income 1,978 Rs in 2004 (Central Bureau of Statistics 2004:37). Government policy stipulates that poor women are entitled to abortion services free of charge, but eligibility criteria have not been clearly defined, and in practice, they tend not to receive any preferential treatment (MOHP and CREHPA 2007; Samandari et al. 2012). Comparisons with the cost of illegal abortions are difficult; five case studies in MOHP et al. (2006) reported considerable variation (200, 500, 700, 3,000, and 8,000 Rs). However, legal abortions are much safer, reducing the likelihood of maternal death and post-abortion complications requiring expensive medical care (MOHP et al. 2006). Consistent with legalization reducing the effective (quality-adjusted) price on an abortion, Valente (2014) showed that having a legal abortion center nearby at the start of a pregnancy reduces the probability of carrying the pregnancy to term by 8.1 %.^10^

In contrast to abortion, contraception services are available free of charge through government facilities; at a subsidized price through social marketing organizations, such as Population Services International; and at full price in private facilities (Shrestha et al. 2012). Condoms, oral contraceptives, and injectables are provided by all levels of government facilities and providers, while IUDs and implants can be obtained in selected hospitals, primary health centers, and health posts (Shrestha et al. 2012). In the latest Demographic and Health Survey (DHS 2011), 55 % (47.5 %) of sterilized women (men) were sterilized in a government hospital or clinic, and 19.4 % (32.5 %) were sterilized through a government-run mobile clinic.

A unique feature of Nepal’s legalization of abortion is its narrow focus. In particular, it was not accompanied by a meaningful increase in the supply of modern contraceptives, an expansion of the reproductive health workforce, or improvements in the provision of other health services. Instead, preexisting reproductive health care providers were trained and licensed to offer abortion services as part of their existing practices.^11^ This feature of Nepal’s policy change allows us to isolate changes in the use of modern contraceptives linked directly to the expansion of abortion supply (and that are not attributable to simultaneous changes in either health service delivery or contraceptive supply, which often accompany such changes in abortion policy (e.g., Pop-Eleches 2010)).^12^

### Trends in Modern Contraceptive Use and Abortion in Nepal

**Fig. 1:**
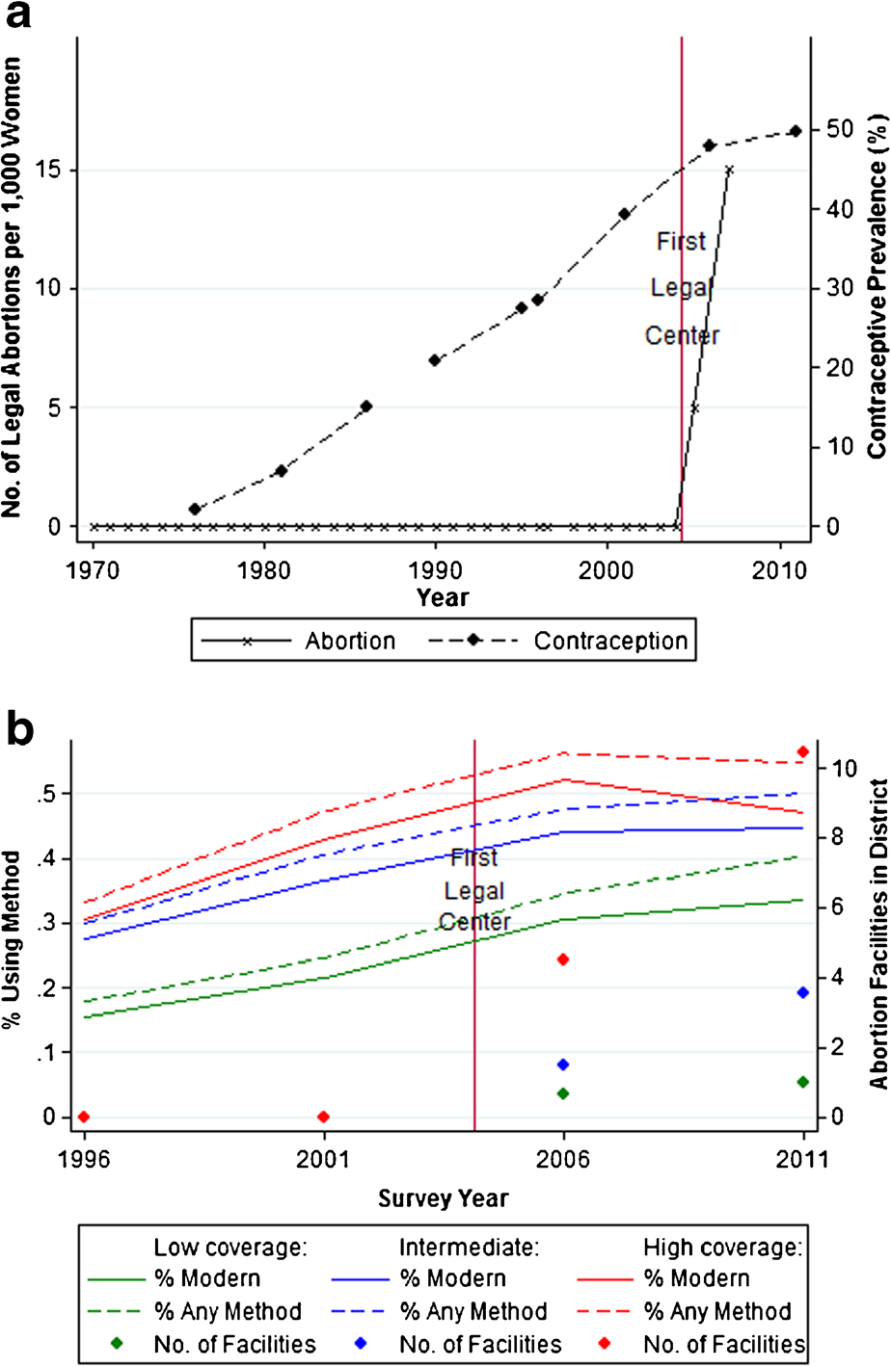
Abortion and contraception trends in Nepal. *Sources:* Panel 1: abortion: Sedgh et al. (2011); contraception: 1970–1987 from Mauldin and Segal (1988), 1990–1995 from United Nations (2004), and 1996–2011 from MOHP et al. (2012). Panel 2: authors’ calculations are based on Demographic and Health Surveys of Nepal (1996–2011) (contraception) and Technical Committee for Implementation of Comprehensive Abortion Care (2010) (abortion facilities)

Figure 1 shows the contraceptive prevalence and abortion rates in Nepal over time. After a rapid, sustained increase in the use of modern contraceptives from the late 1970s until the mid-2000s (from only 2 % to 48 %), contraceptive prevalence then plateaued with the legalization of abortion in 2004 (Fig. 1, panel 1). As in other countries, this pattern of co-movement is consistent with substitution and occurred during a period of declining fertility, with Nepal’s total fertility rate falling from 4.6 in 1996 to 2.6 in 2011 (MOHP et al. 2012).

**Fig. 2:**
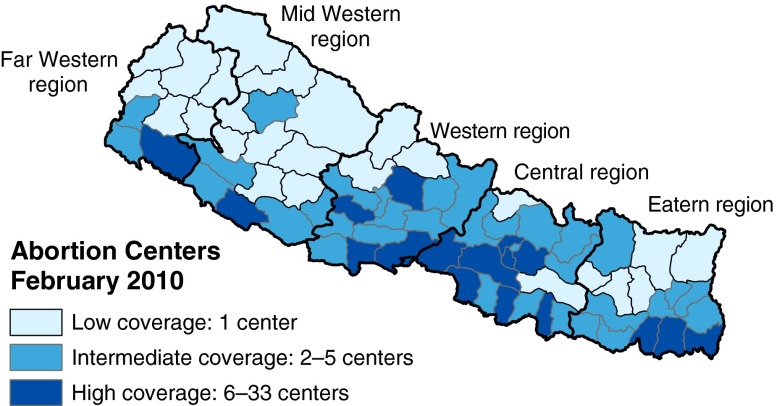
District-level coverage of abortion centers. *Source:* Technical Committee for Implementation of Comprehensive Abortion Care (2010)

However, these aggregate trends may reflect changes in contraceptive use unrelated to the legalization of abortion. A better test of whether the plateauing of contraceptive prevalence is linked to Nepal’s increase in abortion supply would use district-level variation in the magnitude of abortion supply. Figure 2 shows the concentration of legal abortion centers across Nepal’s districts, illustrating substantial geographic variation. Splitting Nepal’s 75 districts into terciles of legal abortion center concentration in 2010, panel 2 of Fig. 1 shows that plateauing in contraceptive prevalence is greater in districts with higher concentrations of legal abortion centers. Panel 2 also shows that areas with fewer abortion centers initially had lower contraceptive prevalence rates. Our estimation strategy accounts for these baseline differences across districts (due to both observable and unobservable, time-invariant factors), assuming that there are no time-varying omitted variables correlated with both the increase in legal abortion centers and contraceptive use. In the Assessment of Robustness and Extensions section, we report a number of robustness tests showing that our results are unlikely to be driven by time-varying omitted variables.

## Conceptual Framework

Before turning to our data and methods used to estimate the relationship between abortion supply and contraceptive use in Nepal, we first present a simple conceptual framework to clarify the hypothesis tested in this article.

Consider the choice between using contraception and not using contraception faced by a woman (couple) who does not want to have a child now (Fig. S2). We define *C*_*i*_, as a dummy variable equal to 1 if woman *i* uses contraception, and 0 otherwise; *A*_*i*_ is a dummy variable equal to 1 if woman *i* has an abortion, and 0 otherwise. Finally, we define *p*_*f*_ as the probability of failure of the contraceptive method used by the woman (and so 0 < *p*_*f*_ < 1). For simplicity, we assume that in the absence of contraception, the woman becomes pregnant with a probability of 1. Assuming a strictly positive probability of less than 1 does not change the qualitative implications of the model, nor does allowing for imperfect predictions of the probabilities of becoming pregnant with and without contraception.^13^ If a woman uses contraception, then with probability 1 – *p*_*f*_, she does not become pregnant and therefore never aborts. With probability *p*_*f*_, she becomes pregnant and either aborts or not. If a woman decides to not use contraception, then she becomes pregnant and either aborts or not.

Now define the costs (financial and psychological) attached to using contraception as *c*^*c*^, the direct costs attached to having an abortion as *c*^*a*^, and the net present value of the costs attached to having an unwanted child as *c*^*u*^; all three variables are allowed to vary across women. Conditional on being pregnant with an unwanted pregnancy, woman *i* aborts if and only if *c*_*i*_^*a*^ < *c*_*i*_^*u*^. Woman *i* will use contraception if and only if her expected cost from using contraception is lower than that from not using contraception:^14^

$$ {c}_i^c<\left(1-{p}_f\right){c}_i^a\ \mathrm{if}\ {c}_i^a<{c}_i^u $$

$$ {c}_i^c<\left(1-{p}_f\right){c}_i^u\ \mathrm{if}\ {c}_i^a\ge {c}_i^u. $$

In summary, a woman will use contraception if and only if

1 $$ {c}_i^c<\left(1-{p}_f\right) \min \left({c}_i^a,{c}_i^u\right). $$

Our hypothesis is that when a legal, safe, and affordable abortion center opens in a woman’s district of residence, *c*_*i*_^*a*^ decreases while all the other parameters of the model remain constant, and hence min(*c*_*i*_^*a*^, *c*_*i*_^*u*^) either decreases or stays the same. Therefore, given that 1 – *p*_*f*_ is positive, Inequality (1) becomes less likely to hold and fewer women use contraception, resulting in substitution of abortion to contraception.

Previous studies estimating the tradeoff between contraceptive use and abortion have generally analyzed how abortion use responds to changes in contraceptive supply. This approach relies heavily on the accuracy of abortion reporting, which is known to be poor in survey data (Jones and Forrest 1992). In contrast, our study investigates how the use of modern contraceptives responds to the provision of legal abortion centers. In doing so, we provide a test of whether women decide not to use contraception up front when it is less difficult/costly to have an abortion (rather than whether they are less likely to have an abortion *ex-post* when the supply of contraceptives increases).

## Data and Methods

### Data on Nepalese Women and Legal Abortion Centers

To measure modern contraceptive use among Nepalese women, we use four waves from the Nepalese Demographic and Health Surveys (DHS): two pre-legalization and two post-legalization (Demographic and Health Surveys of Nepal 1996–2011). Collecting nationally representative data from fertile-age women (defined as ages 15–49) in 1996, 2001, 2006, and 2011, these surveys provide the best available information about reproductive behavior among Nepalese women. Each wave includes a household survey (collecting general information about household composition and socioeconomic characteristics) and an individual survey administered to all fertile-age women, including questions about current and retrospective fertility regulation practices over the preceding four or five years, as well as complete retrospective fertility histories detailing all pregnancies, even those that did not end in a live birth.

We restrict the sample to married women (because the 1996 and 2001 surveys included only married women), but we also assess the robustness of our results to alternative approaches.^15^ A total of 40,622 women were interviewed (8,429 in 1996; 8,726 in 2001; 10,793 in 2006; and 12,674 in 2011). After dropping 2,175 women who are not usual residents of the household in which they are observed, 6,348 unmarried women interviewed in 2006 and 2011, and one woman whose level of education is missing, we obtain the final pooled sample of 32,098 women across the four survey waves.

A brief note about the use of contemporaneous data (from survey years only) versus retrospective contraceptive history data (for years prior to the survey year, as recalled by respondents in survey years) is warranted. An important virtue of using only contemporaneous data is that it minimizes measurement error in reported use of modern contraceptives.^16^ The drawbacks of using only contemporaneous data are the possibility of lower statistical power (because of smaller sample sizes) and less flexibility to examine the evolution of contraceptive use over time relative to the expansion of legal abortion centers. Although we cannot be certain about how much measurement error exists in the retrospective recall data about contraceptive use, studies of contraceptive history recall error have suggested substantial limitations in the use of such recall data (Beckett et al. 2001; Strickler et al. 1997).^17^ Beyond contraceptive use, more recent research has suggested that the quality of recall data deteriorates very rapidly and that the length of the recall period influences self-reported morbidity and use of health services in ways not previously demonstrated (Das et al. 2012).^18^ Given these concerns, the availability of an unusually large number of DHS waves for our analysis (four), and the fact that we have adequate power to examine the correlation between trends in contraceptive use and the intensity of abortion supply (as shown in the Results section), we focus on contemporaneous data in our analysis.

We use the total number of legal abortion centers in each district, month, and year to measure the intensity of abortion supply. We constructed this measure using administrative records from the Nepalese Technical Committee for Implementation of Comprehensive Abortion Care (TCIC 2010) containing exact registration dates for each legal abortion facility authorized before February 2010. We then assign intensity of abortion supply to each woman in our pooled DHS sample at the district-month-year level (according to her interview date).^19^

Table 1 reports descriptive statistics both for our pooled sample and separately for each survey year. The first row reports the mean number of legal abortion centers in the woman’s district in each survey wave. The intensity of abortion supply varies considerably both across survey waves and across districts within each post-legalization wave. On average, women interviewed in 2006 had 2.72 centers in their district (SD = 2.997), and this number rose to 6.34 (SD = 6.702) by 2011.

**Table 1:** Summary statistics

	(1)			(2)			(3)			(4)			(5)		
	DHS 1996			DHS 2001			DHS 2006			DHS 2011			Pooled		
	Mean	SD	*N*	Mean	SD	*N*	Mean	SD	*N*	Mean	SD	*N*	Mean	SD	*N*
A. Abortion Supply and Contraception															
Number of legal abortion centers in district of residence^a^	0.00	0.000	7,496	0.00	0.000	7,842	2.72	2.997	7,776	6.34	6.702	8,984	2.45	4.689	32,098
Any method	0.29		7,496	0.41		7,842	0.50		7,776	0.51		8,984	0.43		32,098
Modern method	0.27		7,496	0.37		7,842	0.46		7,776	0.44		8,984	0.39		32,098
Traditional method	0.02		7,496	0.04		7,842	0.04		7,776	0.07		8,984	0.04		32,098
Modern method other than sterilization	0.09		7,496	0.14		7,842	0.20		7,776	0.21		8,984	0.16		32,098
Female sterilization	0.13		7,496	0.16		7,842	0.19		7,776	0.16		8,984	0.16		32,098
Male sterilization	0.06		7,496	0.07		7,842	0.07		7,776	0.08		8,984	0.07		32,098
Ever had an abortion	0.02		7,496	0.02		7,842	0.04		7,776	0.08		8,984	0.04		32,098
Share of pregnancies aborted^b^	0.00	0.041	6,798	0.00	0.039	7,138	0.01	0.074	7,204	0.03	0.111	8,228	0.01	0.075	29,368
B. Fertility Preferences															
Ideal number of children	2.95	1.059	7,337	2.65	0.879	7,712	2.43	0.830	7,762	2.24	0.788	8,960	2.55	0.927	31,771
C. Covariates															
Urban	0.08		7,496	0.10		7,842	0.15		7,776	0.13		8,984	0.12		32,098
Age	30.58	8.968	7,496	30.95	8.897	7,842	31.47	8.923	7,776	31.68	8.600	8,984	31.20	8.847	32,098
Hindu (excluded category)	0.87		7,496	0.85		7,842	0.86		7,776	0.85		8,984	0.86		32,098
Buddhist	0.06		7,496	0.07		7,842	0.08		7,776	0.08		8,984	0.07		32,098
Muslim	0.05		7,496	0.05		7,842	0.04		7,776	0.04		8,984	0.04		32,098
Christian	0.00		7,496	0.01		7,842	0.01		7,776	0.02		8,984	0.01		32,098
Other religion	0.01		7,496	0.02		7,842	0.01		7,776	0.01		8,984	0.02		32,098
No education (excluded category)	0.80		7,496	0.72		7,842	0.63		7,776	0.49		8,984	0.65		32,098
Primary education	0.11		7,496	0.15		7,842	0.17		7,776	0.19		8,984	0.15		32,098
Secondary education	0.08		7,496	0.12		7,842	0.18		7,776	0.27		8,984	0.16		32,098
Tertiary education	0.01		7,496	0.01		7,842	0.02		7,776	0.06		8,984	0.03		32,098

*Notes:* Statistics are weighted using survey weights. The sample is married women aged 15–49 who usually reside in the household.

*Sources:* Authors’ calculations using Demographic and Health Surveys of Nepal (1996–2011) for all variables except number of legal abortion centers in district of residence, which is based on data from Technical Committee for Implementation of Comprehensive Abortion Care (2010).

^a^Number of legal abortion centers in district of residence is coded using abortion facility data as of February 2010 for the 2011 DHS wave because the administrative records we have had access to end in February 2010.

^b^ Defined only for women with at least one pregnancy.

The next eight rows of Table 1 summarize modern and traditional contraception and abortion. Modern contraceptive use increases between each survey wave until 2006 (from 27 % in 1996 to 46 % in 2006) but then ceases to rise between 2006 and 2011. Among modern methods, the most common is female sterilization, but reversible methods account for most of the increase in contraceptive prevalence between survey waves. In 1996, 2 % of women reported ever having an abortion,^20^ rising to 8 % by 2011. Desired fertility also declined across survey waves. For example, the average ideal number of children fell from 2.95 in 1996 to 2.24 in 2011.^21^

### Statistical Methods

We estimate logit models of the following general form for woman *i* in district *d* observed in survey *s*:

2 $$ \Pr \left({y}_{ids}=1\right)=F\left({\upalpha}_0+\upalpha {C}_{ds}+{\mathbf{X}}_{ids}^{\mathbf{\prime}}\boldsymbol{\upbeta} +{\boldsymbol{\updelta}}_d+{\boldsymbol{\upvarphi}}_s\right), $$

where *F*(*z*) = *e*^*z*^ / (1 + *e*^*z*^) is the cumulative logistic distribution. Here, *y*_*ids*_ is a dichotomous indicator for various measures of contraceptive use (equal to 1 if woman *i* reports using a given method of contraception, and 0 otherwise), *C*_*ds*_ is the number of legal abortion centers in the district at the time of the survey, **X**_*ids*_ is a vector of individual characteristics (urban dummy variable, age, religion dummy variables, education attainment dummy variables), **δ**_*d*_ is a vector of district dummy variables, and **φ**_*s*_ is a vector of (three) DHS wave dummy variables (equivalent to year dummy variables).^22^ We estimate Eq. (2) using survey weights and allowing for error correlation of an arbitrary nature within district.

Equation (2) implements a difference-in-difference estimation strategy in which α captures the effect of each legal abortion center in a woman’s district on contraceptive use, controlling for baseline differences in contraceptive use between districts (**δ**_*d*_) and time trends common to all districts (**φ**_*s*_). The validity of our estimates thus relies on the assumption of no meaningful differences in preexisting fertility regulation trends across districts with varying increases in the supply of legal abortions. In the Assessment of Robustness and Extensions section, we report evidence consistent with this assumption.

## Results

The first six columns of Table 2 report odds ratios estimates of the effect of the number of legal abortion centers (α) for various indicators of contraceptive use (shown at the top of each column) obtained by estimating Eq. (2). The first column shows results for use of any form of contraception (modern or traditional): the addition of a legal abortion center in a woman’s district of residence is associated with a 2.6 % reduction in the odds of using any contraceptive (OR = 0.974; 95 % CI = 0.961, 0.987).^23^ This odds ratio corresponds to a decrease in the probability of using any form of contraception of 0.5 percentage points per legal abortion center (95 % CI = –0.007, –0.002)—implying that a 2 percentage point reduction from the pre-legalization mean of 35 % is associated with four legal abortion centers (the mean number of centers in the two post-legalization survey waves).^24^

**Table 2:** Effect of availability of legal abortion centers on contraceptive use and self-reported abortions

	(1)	(2)	(3)	(4)	(5)	(6)	(7)	(8)
	Any Method	Modern Method	Female Sterilization	Male Sterilization	Modern Method Other Than Sterilization	Traditional Method	Ever Had an Abortion	Share of All Pregnancies Aborted
Number of Abortion Centers in District	0.974**	0.974**	0.978*	0.999	0.976**	0.992	1.013^†^	0.002**
	(0.0065)	(0.0072)	(0.0109)	(0.0106)	(0.0041)	(0.0071)	(0.0080)	(0.0002)
DHS 2001	1.759**	1.678**	1.339**	1.184	1.886**	1.552**	0.879	–0.002*
	(0.1112)	(0.0977)	(0.0817)	(0.1226)	(0.1452)	(0.2408)	(0.1302)	(0.0007)
DHS 2006	2.343**	2.293**	1.699**	1.105	2.681**	1.421*	1.900**	0.000
	(0.2003)	(0.1962)	(0.2224)	(0.1670)	(0.2289)	(0.2276)	(0.3310)	(0.0017)
DHS 2011	2.656**	2.410**	1.591**	1.289	2.987**	2.099**	3.216**	0.007**
	(0.2303)	(0.2249)	(0.2330)	(0.2110)	(0.3140)	(0.3676)	(0.5636)	(0.0025)
Urban	1.324**	1.256**	1.146	1.063	1.264**	1.231**	1.440**	0.009**
	(0.0888)	(0.0876)	(0.1149)	(0.1634)	(0.0801)	(0.0879)	(0.1252)	(0.0022)
Age	1.063**	1.059**	1.080**	1.091**	0.987**	1.026**	1.052**	0.000*
	(0.0030)	(0.0031)	(0.0036)	(0.0043)	(0.0039)	(0.0041)	(0.0054)	(0.0001)
Buddhist	0.679**	0.719**	0.419**	0.640**	1.227	0.735^†^	1.200	0.004
	(0.0533)	(0.0670)	(0.0724)	(0.0925)	(0.1583)	(0.1172)	(0.1800)	(0.0028)
Muslim	0.257**	0.252**	0.112**	0.104**	0.936	0.717	0.504*	–0.005*
	(0.0422)	(0.0427)	(0.0254)	(0.0623)	(0.1824)	(0.2240)	(0.1559)	(0.0026)
Christian	0.804	0.877	0.882	0.744	1.039	0.638	1.095	-0.001
	(0.1776)	(0.1889)	(0.2585)	(0.2481)	(0.1503)	(0.3410)	(0.2868)	(0.0057)
Other	0.498**	0.443**	0.232**	0.446*	0.822^†^	1.223	0.861	–0.000
	(0.0629)	(0.0567)	(0.0502)	(0.1438)	(0.0935)	(0.1891)	(0.1615)	(0.0025)
Primary Education	1.236**	1.158**	0.869^†^	1.658**	1.118^†^	1.497**	2.232**	0.009**
	(0.0700)	(0.0654)	(0.0626)	(0.1596)	(0.0653)	(0.1484)	(0.2263)	(0.0017)
Secondary Education	1.331**	1.114	0.576**	1.352*	1.475**	2.360**	3.250**	0.024**
	(0.0900)	(0.0866)	(0.0703)	(0.1870)	(0.1166)	(0.2763)	(0.4008)	(0.0027)
Tertiary Education	1.305**	0.756*	0.219**	0.651	1.589**	5.381**	3.412**	0.041**
	(0.1344)	(0.0866)	(0.0604)	(0.1912)	(0.1854)	(0.8198)	(0.6233)	(0.0067)
District Dummy Variables Included?	Yes	Yes	Yes	Yes	Yes	Yes	Yes	Yes
Number of Observations	32,098	32,098	31,620	32,078	32,098	31,657	31,371	29,368
Number of Districts	75	75	72	74	75	70	70	75
Pseudo-*R* ^2^	.1102	.1014	.1982	.1604	.0971	.0818	.1327	.0626
Mean Value of Dependent Variable	0.431	0.388	0.158	0.068	0.163	0.044	0.043	0.015

*Notes:* Columns 1–7 report odds ratios from a logit model. Column 8 presents coefficients from a linear regression including a constant (coefficient not reported here). District-correlated robust standard errors in parentheses. Regressions are weighted using survey weights. Sample is married women aged 15–49 who usually reside in the household. Excluded religious category is “Hindu”; excluded education category is “No education.” Some observations are dropped in columns 3, 4, 6, and 7 because of a lack of variation in the value of the dependent variable within district. Observations for women who have never had any pregnancy are dropped in column 8 because the share of aborted pregnancies is not defined for these women.

*Sources:* Authors’ calculations using Demographic and Health Surveys of Nepal (1996–2011) and Technical Committee for Implementation of Comprehensive Abortion Care (2010).

^†^ *p* < .10; *p < .05; **p < .01

Columns 2 and 6 report separate estimates for use of any modern and any traditional method of contraception, respectively.^25^ The odds of using modern contraceptives decrease by 2.6 % with an additional abortion center, while the odds ratio for use of traditional methods is indistinguishable from one (OR = 0.974; 95 % CI = 0.960, 0.989; and OR = 0.992; 95 % CI = 0.978, 1.006, respectively). Taken together, these results suggest that when a legal abortion facility opens in a woman’s district, she reduces her use of modern contraceptives, while traditional contraception remains unchanged. Analyzing the effect of an additional abortion center on modern contraceptive use by age group, we find the largest decrease in contraceptive use among those aged 15–19 and 30–34, and the effect is statistically significant for all groups up to 35–39 (Online Resource 1, Table S1).

Columns 3, 4, and 5 of Table 2 analyze how substitution away from modern contraception with the opening of legal abortion centers varies between sterilization and reversible modern methods.^26^ Column 3 shows that an additional abortion center is associated with a 2.2 % reduction in the odds of female sterilization (OR = 0.978; 95 % CI = 0.957, 0.999), implying a 0.23 percentage point decrease in the prevalence of female sterilization. On the contrary, we find that abortion centers have no effect on male sterilization (column 4). The estimated change in odds of using reversible modern methods reported in column 5 is similar to that of using female sterilization, declining by 2.4 % with each additional legal abortion facility (OR = 0.976; 95 % CI = 0.968, 0.984).^27^

If our interpretation of the estimates in the first six columns of Table 2 is correct, the expansion of legal abortion centers should also be associated with an increase in the probability that women abort (although an effect on contraceptive use may be detected before the effect on abortion is realized). The seventh column of Table 2 reports results obtained by reestimating Eq. (2) using a dichotomous indicator for whether a woman reports ever having an abortion (defined as a pregnancy that did not result in a live birth and for which someone has done something to end the pregnancy). Each additional legal abortion center in a woman’s district is associated with a 1.3 % increase in odds of ever having an abortion, which is statistically significant at the 90 % level (OR = 1.013; 95 % CI = 0.998, 1.029), and implies a 4 % increase relative to the pre-legalization proportion reporting ever having an abortion for four legal abortion centers. Because the likelihood of ever having an abortion partly depends on the number of past pregnancies, we confirm that the estimates in column 7 of Table 2 are not driven by changes in fertility by using the share of pregnancies aborted by the respondent as the dependent variable (estimating a linear specification by ordinary least squares). Column 8 shows that the abortion center estimate is again positive and statistically significant (linear coefficient = 0.0019; 95 % CI = 0.0015, 0.0022).

## Assessment of Robustness and Extensions

### Testing for Preexisting Trend Differences

Although our difference-in-difference estimation framework accounts for baseline differences in contraceptive prevalence across districts, it assumes that districts with varying concentrations of abortion facilities had parallel trends in contraceptive prevalence prior to the legalization of abortion. To test whether the number of abortion centers was targeted to districts with preexisting trend differences in contraceptive prevalence, we conduct two related placebo experiments.

In the first, we assign a district-level measure of the future number of abortion centers (the number of centers at the time of next survey) to each woman in the 1996 and 2001 DHS waves (i.e., before any legal abortion center opened). Reestimating Eq. (2) using future number of abortion centers in lieu of the current number of centers, Table 3 reports estimates for the parameter α′ in the equation *Pr*(*y*_*ids*_ = 1) = *F*(α_0_^′^ + α′*C*_*ds* + 1_ + **X**_*ids*_^′^**β**′ + **δ**_*d*_^′^ + **φ**_*s*_^′^). Consistent with our assumption of parallel trends, none of these estimated odds ratios are significantly different from 1, nor is the estimate for future number of abortion centers estimated by ordinary least squares (OLS) in column 8 of Table 2 significantly different from 0.

**Table 3:** Control experiment 1: Effect of availability of future legal abortion centers before any center opened

	(1)	(2)	(3)	(4)	(5)	(6)	(7)	(8)
	Any Method	Modern Method	Female Sterilization	Male Sterilization	Modern Method Other Than Sterilization	Traditional Method	Ever Had an Abortion	Share of All Pregnancies Aborted
Number of Abortion Centers at Next Survey Date	1.011	1.004	1.000	0.928	0.994	0.986	0.967	-0.000
	(0.0386)	(0.0341)	(0.0220)	(0.0453)	(0.0190)	(0.0355)	(0.0243)	(0.0002)
Number of Observations	15,338	15,338	14,324	15,338	15,310	14,601	12,994	13,936
Number of Districts	72	72	61	72	71	63	50	72
Pseudo-*R* ^2^	.1243	.1219	.1698	.1640	.1198	.0800	.0749	.0207
Mean Value of Dependent Variable	0.352	0.319	0.149	0.062	0.116	0.034	0.019	0.005

*Notes:* Data are omitted for the following variables: dummy variable for DHS 2001; district fixed effects; and controls for urban location, age at interview, religion, and education summarized in Table 1, panel C. Columns 1–7 report odds ratios from a logit model. Column 8 presents coefficients from a linear regression including a constant (coefficient not reported here). District-correlated robust standard errors are shown in parentheses. Regressions are weighted using survey weights. The sample is married women aged 15–49 who usually reside in the household. Some observations are dropped in columns 3, 4, 6, and 7 because of lack of variation in the value of the dependent variable within district. Observations for women who have never had any pregnancy are dropped in column 8 because the share of aborted pregnancies is not defined for these women.

*Sources:* Authors’ calculations using Demographic and Health Surveys of Nepal (1996, 2001) and Technical Committee for Implementation of Comprehensive Abortion Care (2010).

The second placebo experiment repeats the first with two differences: it uses data from the 2006 DHS wave, and it includes both current and future number of legal abortion facilities (because some centers were operating in 2006). Table 4 shows estimates for future and current number of legal abortion facilities, again suggesting that current contraceptive prevalence and past abortion behavior are not correlated with future abortion supply. Overall, these results suggest no targeting of abortion centers to districts with preexisting trend differences in contraceptive prevalence, and they are consistent with our interpretation of Table 2, showing evidence that abortion and the use of modern contraceptives are substitutes.

**Table 4:** Control experiment 2: Effect of availability of future legal abortion centers over and above the effect of current availability

	(1)	(2)	(3)	(4)	(5)	(6)	(7)	(8)
	Any Method	Modern Method	Female Sterilization	Male Sterilization	Modern Method Other Than Sterilization	Traditional Method	Ever Had an Abortion	Share of All Pregnancies Aborted
Number of Abortion Centers	0.928**	0.921**	0.909*	0.969	0.966	1.043	1.057	0.001
	(0.0257)	(0.0291)	(0.0374)	(0.0783)	(0.0223)	(0.0469)	(0.0609)	(0.0009)
Number of Abortion Centers at Next Survey Date	1.020	1.022	1.035	0.970	1.001	0.982	0.958^†^	-0.000
	(0.0173)	(0.0177)	(0.0235)	(0.0372)	(0.0104)	(0.0231)	(0.0245)	(0.0004)
Number of Observations	23,114	23,114	22,343	23,063	23,114	22,730	22,260	21,140
Number of Districts	75	75	68	73	75	69	66	75
Pseudo-*R* ^2^	.1215	.1173	.1971	.1546	.1144	.0692	.1003	.0318
Mean Value of Dependent Variable	0.400	0.366	0.161	0.063	0.146	0.035	0.026	0.008

*Notes:* Data are omitted for the following variables: two dummy variables for DHS 2001 and 2006; district fixed effects; and controls for urban location, age at interview, religion, and education summarized in Table 1 Panel C. Columns 1–7 report odds ratios from a logit model. Column 8 presents coefficients from a linear regression including a constant (coefficient not reported here). District-correlated robust standard errors are shown in parentheses. Regressions are weighted using survey weights. The sample is married women aged 15–49 who usually reside in the household. Some observations are dropped in columns 3, 4, 6, and 7 because of lack of variation in the value of the dependent variable within district. Observations for women who have never had any pregnancy are dropped in column 8 because the share of aborted pregnancies is not defined for these women.

*Sources*: Authors’ calculations using Demographic and Health Surveys of Nepal (1996–2006) and Technical Committee for Implementation of Comprehensive Abortion Care (2010).

^†^ *p* < .10; *p < .05; **p < .01

### Other Robustness Tests

For completeness, we also estimate variants of Eq. (2) using recall data contained in the 2006 and 2011 DHS fertility histories and report our results in Online Resource 1, Table S2. Our specifications use woman-month observations from April 2000 to February 2010 and exclude women who were sterilized or whose husbands were sterilized by March 2004; *C*_*ds*_ is replaced by *C*_*dm*_, the number of legal abortion centers in the district for each month and year. We find a negative, statistically significant relationship between the number of abortion centers in a woman’s district and her odds of reporting use of any contraceptive method, confirming our inferences from contemporaneous data (column 1). This estimate is robust to controlling for linear, quadratic, or cubic district-specific trends (columns 2, 3, and 4, respectively). When we add a placebo treatment variable equal to the number of abortion centers in the district 12 months in the future, the result persists, and the effect of the placebo treatment variable is statistically insignificant (column 5).

We then investigate the robustness of our main results to addressing a variety of other potential concerns:

1. First, we control for a number of additional regressors in panel A of Table 5. Specifically, we control for respondents’ ideal number of children; number of conflict casualties in the year preceding the survey in respondents’ districts (per 1991 district population, the year of the last pre-conflict population census); whether respondents reported having heard a family planning message on the radio in the last month; whether respondents were visited by a family planning worker in the previous 12 months; whether respondents had heard of AIDS; and socioeconomic status (measured by quintile in the distribution of household asset ownership). Our conclusions do not change after we include these additional controls.^28^
2. Second, in panel B, we restrict the sample analyzed in panel A to women who were not sterilized and whose husbands were not sterilized as of March 2004. The results confirm the sign, significance, and magnitude of the main estimates for all modern contraception and for temporary methods.^29^

**Table 5:** Robustness checks

	(1)	(2)	(3)	(4)	(5)	(6)	(7)	(8)
	Any Method	Modern Method	Female Sterilization	Male Sterilization	Modern Method Other Than Sterilization	Traditional Method	Ever Had an Abortion	Share of All Pregnancies Aborted
A. Include Further Controls (see notes for details)								
Number of abortion centers	0.980**	0.980**	0.983^†^	1.001	0.981**	0.996	1.014^†^	0.002**
	(0.0055)	(0.0062)	(0.0100)	(0.0089)	(0.0041)	(0.0074)	(0.0073)	(0.0002)
Number of observations	31,762	31,762	31,288	31,743	31,762	31,325	31,045	29,063
B. Further Controls + Restrict Sample to Nonsterilized Couples as of March 2004 (results in columns 3 and 4 restricted to DHS 2006 and 2011)								
Number of abortion centers	0.973**	0.970**	0.987	1.141^†^	0.977**	0.994	1.011	0.002**
	(0.0058)	(0.0067)	(0.0265)	(0.0833)	(0.0044)	(0.0073)	(0.0076)	(0.0002)
Number of observations	25,890	25,890	12,095	12,026	25,890	25,517	25,264	23,214
C. As Panel B + Scale Number of Abortion Centers by District Population								
Number of abortion centers	0.983	0.976^†^	1.001	1.063^†^	0.982*	1.007	1.012	0.001**
	(0.0106)	(0.0122)	(0.0238)	(0.0380)	(0.0083)	(0.0134)	(0.0104)	(0.0004)
Number of observations	25,890	25,890	12,095	12,026	25,890	25,517	25,264	23,214
D. As Panel B + Allow for Time Trends to Vary by District Population								
Number of abortion centers	0.965**	0.955*	0.991	1.102	0.979*	1.015	1.030*	0.002**
	(0.0125)	(0.0173)	(0.0366)	(0.0932)	(0.0083)	(0.0156)	(0.0142)	(0.0004)
Number of observations	25,890	25,890	12,095	12,026	25,890	25,517	25,264	23,214
E. As Panel B + Allow Time Trends to Vary by Region								
Number of abortion centers	0.965**	0.960**	0.941	1.142	0.971**	1.009	1.018	0.002**
	(0.0077)	(0.0094)	(0.0491)	(0.1005)	(0.0094)	(0.0128)	(0.0166)	(0.0003)
Number of observations	25,890	25,890	12,095	12,026	25,890	25,517	24,898	23,214
F. As Panel B + Allow Time Trends to Differ in Rural and Urban Areas								
Number of abortion centers	0.977**	0.976**	0.994	1.142	0.977**	0.993	1.017*	0.001**
	(0.0059)	(0.0063)	(0.0245)	(0.0950)	(0.0050)	(0.0086)	(0.0084)	(0.0003)
Number of observations	25,890	25,890	12,095	12,026	25,890	25,517	25,264	23,214
G. As Panel B + Allow Time Trends to Differ by Wealth Quintile								
Number of abortion centers	0.986*	0.986^†^	0.974	1.149^†^	0.984**	0.994	1.011	0.001**
	(0.0068)	(0.0072)	(0.0264)	(0.0965)	(0.0044)	(0.0088)	(0.0082)	(0.0002)
Number of observations	25,890	25,890	12,095	12,026	25,890	25,517	25,264	23,214

*Notes:* Data are omitted for the following variables: three dummy variables indicating DHS waves; district fixed effects; and controls for urban location, age at interview, religion, education, ideal number of children, control for the number of conflict casualties in the year preceding the survey (per district population as of 1991, the last preconflict population census), whether the woman reports having heard a family planning message on the radio in the last month, whether she was visited by a family planning worker in the previous 12 months, whether she has heard of AIDS, and the SES group to which she belongs (as measured by the her quintile in the distribution of household living standard). Columns 1–7 report odds ratios from a logit model. Column (8) presents coefficients from a linear regression including a constant (coefficient not reported here). District-correlated robust standard errors are shown in parentheses. Regressions are weighted using survey weights. Sample is of married women aged 15–49 who usually reside in the household. Some observations are dropped in columns 3, 4, 6, and 7 because of lack of variation in the value of the dependent variable within district. In panels B–F, columns 3 and 4 exclude observations for 1996 and 2001 because by definition, there is no variation in sterilization status in these surveys after dropping those sterilized before March, 2004. Observations for women who have never had any pregnancy are dropped in column 8 because the share of aborted pregnancies is not defined for these women.

*Sources:* Authors’ calculations using Demographic and Health Surveys of Nepal (1996–2011) and Technical Committee for Implementation of Comprehensive Abortion Care (2010).

^†^ *p* < .10; *p < .05; **p < .01
3. Third, in panel C, we further scale the number of abortion centers by district population as of 2001, the date of the last pre-legalization population census. Our estimates become more imprecise (the standard errors nearly double), but the negative association between legal abortion centers and the prevalence of any modern contraception—and, specifically, temporary methods—remains statistically significant.^30^
4. Fourth, in panels D, E, F, and G, we explicitly allow time trends to vary by pre-legalization district population (panel D), region (panel E),^31^ rural/urban location (panel F), and wealth quintile (panel G). More populous districts, districts in the more accessible regions of the country, and urban areas experienced earlier/more intense expansions of legal abortion supply. Additionally, private providers are more prevalent in urban areas, and these private providers may be more responsive to local demand than public facilities. The two main national health and population programs in place during the relevant period (the Nepal Family Health Program during 2001–2006 and the Nepal Health Sector Program Implementation Plan during 2004–2009) also aimed to prioritize the poor and those living in remote areas (MOHP et al. 2012). Interacting DHS wave and initial population, region, urban location, and wealth quintile in panels D, E, F, and G (respectively) show that our conclusions are unchanged when allowing for systematic trend differences in contraceptive use by these characteristics.

Finally, we explore the robustness of our conclusions to a variety of weighting, functional form, and sample considerations. We find that our results are robust to using unweighted rather than weighted logit models (panel A of Table 6), to replacing our logit specification with a linear probability model (panel B of Table 6), to excluding each DHS survey in turn to investigate whether our conclusions depend on any individual survey (Table 7),^32^ and to limiting the sample to all women ages 25–49 instead of restricting our sample to married women (Table 8).^33^

**Table 6:** Additional robustness checks

	(1)	(2)	(3)	(4)	(5)	(6)	(7)	(8)
	Any Method	Modern Method	Female Sterilization	Male Sterilization	Modern Method Other Than Sterilization	Traditional Method	Ever Had an Abortion	Share of All Pregnancies Aborted
A. Unweighted Regressions								
Number of abortion centers	0.968**	0.967**	0.979*	0.987	0.972**	0.995	1.003	0.002**
	(0.0058)	(0.0063)	(0.0096)	(0.0109)	(0.0043)	(0.0067)	(0.0067)	(0.0002)
Number of observations	32,098	32,098	31,620	32,078	32,098	31,657	31,371	29,368
B. Linear Probability Model								
Number of abortion centers	–0.005**	–0.005**	–0.002*	0.000	–0.003**	–0.000	0.003**	
	(0.0016)	(0.0018)	(0.0011)	(0.0008)	(0.0007)	(0.0004)	(0.0006)	
Number of observations	32,098	32,098	32,098	32,098	32,098	32,098	32,098	

*Notes:* Data are omitted for the following variables: three dummy variables indicating DHS waves; district fixed effects; and controls for urban location, age at interview, religion, and education summarized in Table 1, panel C. See also the footnotes to Table 2.

*p < .05; **p < .01

**Table 7:** Robustness of the effect of abortion centers to excluding one survey at a time

	(1)	(2)	(3)	(4)	(5)	(6)	(7)	(8)
Excluded Data	Any Method	Modern Method	Female Sterilization	Male Sterilization	Modern Method Other Than Sterilization	Traditional Method	Ever Had an Abortion	Share of All Pregnancies Aborted
DHS 1996	0.975**	0.977**	0.980^†^	1.024^†^	0.977**	0.993	1.024*	0.002**
	(0.0065)	(0.0073)	(0.0120)	(0.0140)	(0.0048)	(0.0093)	(0.0097)	(0.0002)
	24,602	24,602	24,228	24,202	24,602	24,238	24,056	22,570
DHS 2001	0.976*	0.976*	0.979^†^	0.993	0.977**	0.990	1.013^†^	0.002**
	(0.0110)	(0.0107)	(0.0122)	(0.0158)	(0.0055)	(0.0076)	(0.0080)	(0.0002)
	24,256	24,256	23,846	23,902	24,256	23,529	23,684	22,230
DHS 2006	0.974**	0.974**	0.981^†^	0.996	0.974**	0.990	1.004	0.002**
	(0.0070)	(0.0077)	(0.0109)	(0.0111)	(0.0043)	(0.0074)	(0.0079)	(0.0002)
	24,322	24,322	23,858	24,322	24,322	23,810	23,422	22,164
DHS 2011	0.957**	0.951**	0.953^†^	0.924**	0.968**	1.016	0.986	0.000
	(0.0123)	(0.0144)	(0.0239)	(0.0245)	(0.0104)	(0.0212)	(0.0260)	(0.0005)
	23,114	23,114	22,343	23,063	23,114	22,730	22,260	21,140

*Notes:* Data are omitted for the following variables: two dummy variables indicating DHS waves; district fixed effects; and controls for urban location, age at interview, religion, and education summarized in Table 1, panel C. See also the footnotes to Table 2.

^†^ *p* < .10; *p < .05; **p < .01

**Table 8:** Robustness of the effect of abortion centers to including all interviewed women aged ≥25 instead of restricting 2006 and 2011 surveys to ever-married women

	(1)	(2)	(3)	(4)	(5)	(6)	(7)	(8)
	Any Method	Modern Method	Female Sterilization	Male Sterilization	Modern Method Other Than Sterilization	Traditional Method	Ever Had an Abortion	Share of All Pregnancies Aborted
Number of Abortion Centers	0.977**	0.979**	0.979^†^	1.001	0.977**	0.988	1.007	0.002**
	(0.0051)	(0.0060)	(0.0106)	(0.0099)	(0.0044)	(0.0078)	(0.0092)	(0.0003)
Number of Observations	25,174	25,174	24,843	25,150	25,174	24,848	24,454	24,268
Number of Clusters	75	75	72	74	75	70	69	75
Pseudo-*R* ^2^	.0665	.0618	.1607	.1177	.1006	.0828	.1259	.0736
Mean Y	0.479	0.435	0.196	0.085	0.155	0.045	0.050	0.016

*Notes:* Data are omitted for the following variables: three dummy variables indicating DHS waves; district fixed effects; and controls for urban location, age at interview, religion, and education. Columns 1–7 report odds ratios from a logit model. Column 8 presents coefficients from a linear regression including a constant (coefficient not reported here). District-correlated robust standard errors are shown in parentheses. Regressions are weighted using survey weights. The sample is women aged 25–49 who usually reside in the household, irrespective of their marital status. The excluded religious category is “Hindu,” and the excluded education category is “No education.” Some observations are dropped in columns 3, 4, 6, and 7 because of lack of variation in the value of the dependent variable within district. Observations for women who have never had any pregnancy are dropped in column 8 because the share of aborted pregnancies is not defined for these women.

*Sources:* Authors’ calculations using Demographic and Health Surveys of Nepal (1996–2011) and Technical Committee for Implementation of Comprehensive Abortion Care (2010).

^†^ *p* < .10; **p < .01

### Consideration of Changes in Temporary Modern Methods Versus Sterilization

The results presented so far suggest that the increase in the supply of legal abortions affected the use of temporary modern contraceptive methods, but its effect on new sterilizations is less clear. One plausible explanation for reductions in the cost of abortion to affect temporary contraception but not sterilization can be understood by returning to our conceptual framework presented earlier. Rearranging Inequality (1) dividing each side by 1 – *p*_*f*_ and allowing for more than one type of contraceptive method denoted by *m*, woman *i* will choose the contraceptive method with the lowest perceived ratio of cost to success rate (*c*_*im*_^*c*^ / 1 − *p*_*fm*_) as long as the value of this ratio is less than min(*c*_*i*_^*a*^, *c*_*i*_^*u*^). If changes in abortion supply affect the decisions of only those women for whom the perceived ratio of cost to success (*c*_*i*_^*c*^/1 − *p*_*f*_) is higher for sterilization than for temporary methods, the relevant tradeoff is between temporary methods and no contraception. This could be the case if women who face a high cost of having an abortion regardless of whether it is legally and safely provided—because of moral considerations or high transport costs, for example—were also more likely to have a lower perceived cost-to-success rate of sterilization relative to temporary methods. If this were the case, then legal abortion centers would not decrease *c*_*i*_^*a*^ sufficiently to affect the contraception choice of women who would choose sterilization over temporary methods.

## Conclusion

Although scholars have written extensively about the relationship between the use of modern contraceptives and abortion and have generally reported an inverse relationship between the two, a causal relationship has been difficult to isolate. A key difficulty is the fact that reproductive health programs often alter many aspects of service delivery simultaneously—expanding the reproductive health workforce, bundling new contraception and abortion services, and improving the quality of health services generally. Even the famous Matlab Family Planning Experiment integrated the provision of modern contraceptives with the provision of both abortion services (menstrual regulation) and antenatal and child health services, making it difficult to isolate the effect of contraceptive supply.

This study analyzes the relationship between contraceptive use and abortion during the rapid scale-up of legal abortion services across Nepal—a “natural experiment” in which abortion services were not accompanied by changes in contraceptive supply or other potentially confounding health policy changes. Using four DHS survey waves (two before and two after legalization) and an official census of all legal abortion centers, we find that each legal abortion center in a woman’s (couple’s) district of residence was associated with a 2.6 % reduction in the odds of using any contraceptive. For the mean number of centers per district in the post-legalization period (four), our estimates imply that Nepal’s expansion of abortion supply was associated with a 2 percentage point decline in the use of contraceptives—a 6 % decrease relative to the pre-legalization mean.^34^ This decline in contraceptive use occurs among modern (but not traditional) methods and is driven most robustly by changes in the use of reversible modern methods (primarily injections and, to a lesser extent, condoms and the pill). Our direct assessments of the parallel trends assumption underlying our difference-in-difference study design also strengthens the interpretation that our estimates provide evidence of true substitution between use of modern contraceptives and abortion.

We emphasize two important policy implications of our findings. First, policies aiming to reduce the full cost of abortion (e.g., financial, social, psychological) should be accompanied by measures to reduce the full cost of contraceptive use (broadly defined to include social and psychological costs) if policymakers wish to avoid substitution from contraception to abortion. Second, in demonstrating a tradeoff between contraception and abortion, our findings also suggest that reductions in the cost of contraception may reduce the incidence of abortion.

## Electronic supplementary material

Below is the link to the electronic supplementary material.

ESM 1 (DOCX 556 kb)

## Footnotes

1 Because we study married women in a patriarchal society, the choice of contraception is likely to be the result of intrahousehold bargaining. We do not theoretically or empirically distinguish individual preferences from the choices that result from this bargaining process. However, our reduced-form estimates isolate important—and policy-relevant—parameters of interest. Recognizing this point, we refer to contraceptive decisions as women’s decisions for simplicity throughout the article.

2 A separate strand of economic theory, which studies the response of risk-taking behavior to perceived changes in the consequences of a bad outcome (e.g., increased automobile safety, availability of treatment for a medical condition), suggests an additional mechanism through which women who do not want to become pregnant may reduce contraceptive use when access to abortion improves. Indeed, evidence suggests that reducing the cost of a bad outcome may increase risk-taking in other health areas. Examples are Peltzman (1975) on the effect of automobile safety on dangerous driving, Dilley et al. (1997) on HIV treatment and risk-taking among men who have sex with men, and Peltzman (2011) on the effect of medical breakthroughs (e.g., new treatments for heart disease) on offsetting behavior (e.g., obesity).

3 The theoretical discussion of the tradeoff between contraception and abortion among demographers has tended to focus on the effect of changing contraceptive prevalence on the abortion rate (e.g., Bongaarts and Westoff 2000). In the economics literature, the tradeoff is understood as going both ways (for a discussion of the effect of a change in the cost of abortion on contraceptive use, see Kane and Staiger 1996). The difference stems from the economic modeling of contraceptive choices as depending on the cost of contraception relative to the cost of not using contraception, which in turn depends on the cost of abortion (see the Conceptual Framework section).

4 Changes in supply include both changes in availability and changes in the full price of contraception (monetary, social, and emotional price). For brevity, we refer to these changes collectively as “supply changes” throughout.

5 First announced in Mexico City in 1984 by President Ronald Reagan’s administration, the Mexico City Policy requires all non-governmental organizations (NGOs) operating abroad to refrain from performing or counseling women about abortion as a means of fertility control as a condition for receiving U.S. federal funding.

6 *Menstrual regulation* refers to manual vacuum aspiration procedures conducted after a missed menstrual period but before pregnancy is clinically confirmed.

7 In 1920, the Soviet Union was the first country to legalize abortion.

8 Although imprisonment was not a common outcome among women who had an abortion, among the small population of female inmates in Nepalese prisons (405 in 1997), a substantial proportion are believed to have been convicted on abortion-related charges (Ramaseshan 1997).

9 A total of 291 abortion centers in 2010 relative to a total Nepal population of 26.49 million reported in the 2011 Nepalese population census implies one center per 91,031 inhabitants. In the United States, there were 1,793 abortion providers in 2008 (Guttmacher Institute 2013) relative to a total population of 305 million (Population Reference Bureau 2008), implying one provider per 170,106 inhabitants.

10 This figure is based on a binary definition of proximity to a legal abortion center corresponding to the median distance to the nearest legal abortion center (28.6 kilometers). Various robustness checks for different definitions of access to a legal abortion center are presented in Valente (2014).

11 A survey of Comprehensive Abortion Care (CAC) providers conducted in 2009 revealed that only 8 of 139 surveyed providers introduced new contraceptive services or maternal and child health services at approximately the same time as they started providing abortion services (Valente 2014).

12 Of course, most abortion clients receive post-abortion contraceptive counseling (MOHP and CREHPA 2006). This could lead us to underestimate the extent of the substitution away from contraception if women who use abortion services are more likely to use contraception after having had an abortion. However, the results of our statistical analysis are virtually unchanged when excluding women who report having had an abortion within one year of the survey, thus suggesting that post-abortion changes in contraceptive use are not influencing our findings (full results are available on request).

13 The only difference when assuming a probability of becoming pregnant in the absence of contraception inferior to one is that Inequality (1) becomes *c*_*i*_^*c*^ < *p*_*p*_(1 − *p*_*f*_)min(*c*_*i*_^*a*^, *c*_*i*_^*u*^), where *p*_*p*_ is the probability of becoming pregnant in the absence of effective contraception, and *p*_*f*_ is the probability of becoming pregnant despite using contraception when contraceptive protection is needed (which is the case with probability *p*_*p*_). Allowing women to hold erroneous beliefs in terms of *p*_*p*_ and *p*_*f*_ changes the ranges of costs over which women decide to use contraception or not, but it does not alter the qualitative conclusions of the model about the effect of a decrease in the cost of abortion relative to contraception.

14 When *c*_*i*_^*a*^ < *c*_*i*_^*u*^, the expected cost of using contraception is *p*_*f*_(*c*_*i*_^*c*^ + *c*_*i*_^*a*^) + (1 − *p*_*f*_)*c*_*i*_^*c*^ = *p*_*f*_*c*_*i*_^*a*^ + *c*_*i*_^*c*^, and the cost of not using contraception is *c*_*i*_^*a*^. When *c*_*i*_^*a*^ ≥ *c*_*i*_^*u*^, the expected cost of using contraception is *p*_*f*_(*c*_*i*_^*c*^ + *c*_*i*_^*u*^) + (1 − *p*_*f*_)*c*_*i*_^*c*^ = *p*_*f*_*c*_*i*_^*u*^ + *c*_*i*_^*c*^, and the cost of not using contraception is *c*_*i*_^*u*^.

15 For example, we also restrict our sample to women ages 25 and older, among whom marriage is nearly universal: 97.3 % of respondents ages 25 and above in the 2006 and 2011 surveys were married at the time of interview. Focusing on married women is consistent with the composition of legal abortion service clients in Nepal. A survey conducted by CREHPA in 2006 indicated that nearly 98 % of clients were or had been married, close to 60 % were aged 20–29 (only 5 % were under 20), and less than 7 % had no living child (23 %, 40 %, and 31 % had one, two, and three or more living children, respectively) (MOHP and CREHPA 2007). An international comparison of legal abortion service client characteristics suggests that the characteristics of Nepalese clients are generally comparable with those observed in other less-developed countries (Bankole et al. 1999, MOHP and CREHPA 2007).

16 The question asked in the DHS is, “Are you currently doing something or using any method to delay or avoid getting pregnant?”

17 Strickler et al. (1997) and Beckett et al. (2001) compared contraceptive use by the same woman for the same periods of time but reported at two different survey dates. They found substantial inconsistencies at the disaggregated level. Specifically, Strickler et al. (1997) found that among Moroccan women reporting at least one period of contraceptive use, only 45.1 % reported periods of use and non-use in the same order in two different surveys, and only 29.3 % reported the same sequence and length of contraceptive use. Similarly, when comparing individual reports of contraceptive use in contemporaneous and recall data for Malaysia, Beckett et al. (2001) obtained a kappa coefficient of only 0.38. In the presence of misclassification (e.g., reporting not using contraception when in fact using and vice versa), Hausman et al. (1998) showed that estimates are inconsistent and that their precision can be overstated.

18 Recall error for the variables Das et al. (2012) examined, which include visits to the doctor and self-medication, is very relevant to recall error in contraceptive use but less so for more salient events, such as the birth of a child.

19 By definition, an abortion center is legal only if it is listed with the Technical Committee for Implementation of Comprehensive Abortion Care (TCIC), and the data used here are based on the list of all facilities included on the TCIC list up to February 2010.

20 For each pregnancy in the pregnancy history of the woman, she is asked whether the baby [was] “born alive, born dead, or lost before birth”; when the pregnancy ended and how long it lasted; and then “did you [the respondent] or someone else do something to end this pregnancy?” The sequence of questions is the same for the four surveys, but the variables available changed in 2011. Before 2011, we count as an abortion any pregnancy not ending in live birth for which the woman either says that something was done to end the pregnancy or refuses to answer the last question. In the 2011 survey, the data set does not contain the necessary raw data to apply the same rule, but contrary to the previous surveys, provides a classification of pregnancies as live birth, stillbirth, miscarriage, or abortion based on the same survey questions.

21 Table 1, panel C, shows that our sample is predominantly rural, with low levels of education (especially among women) that increase rapidly across study waves.

22 Note that *C*_*ds*_ is coded using abortion facility data as of February 2010 for the 2011 DHS wave because the administrative records for which we had access end in February 2010.

23 This and the other point estimates of interest in Table 2 are nearly unchanged when the linear age variable is replaced with seven 5-year age categories. Results are available on request.

24 Marginal effects reported in this article are computed at the mode of all categorical covariates, the mean of maternal age, and the mean number of abortion centers per district in the two post-legalization surveys.

25 Traditional methods, such as withdrawal and the rhythm method, are used by only 4 % of women in our pooled sample; however, their use has increased over time, from 2 % in 1996 to 7 % in 2011.

26 Among reversible modern methods, condoms may require more negotiation with male partners. Repeating our estimation separately for condom use (OR = 0.989; 95 % CI = 0.979, 1.0004) and for other reversible methods (OR = 0.976; 95 % CI = 0.967, 0.984), we find a larger association for other reversible modern methods, although the difference between them is not statistically significant.

27 Odds ratios on the other covariates generally have the expected signs: the indicators for each DHS survey capture the overall trends in contraceptive use described in the section Data on Nepalese Women and Legal Abortion Centers and confirm that urban, better-educated, and older women are more likely to use contraception. Coefficients on religious affiliation variables are also reasonable (e.g., Muslims are significantly less likely to use contraception than Hindus). It is interesting to note that the education gradient is steep for traditional methods but much less so for use of any type of contraception.

28 We also estimate the robustness test shown in Table 4, panel A, including both the number of children born to a woman and whether a woman had a job in the past 12 months. The resulting estimates change very little (and insignificantly so) when we include these additional covariates. These results are available upon request.

29 In this restricted sample, by definition, there is no variation in sterilization status in the 1996 and 2001 DHS (because those sterilized by 1996 or 2001 are dropped from the sample), so that we can use only the 2006 and 2011 DHS surveys for the analysis of the sterilization outcomes. The findings on sterilization outcomes using only the last two surveys suggest a statistically insignificant decrease in female sterilization and a marginally significant increase in male sterilization, although the total effect on modern contraception is still significantly and consistently negative overall.

30 Although results are less precise, the number of abortion centers per inhabitant is very similar to our main measure of program treatment: the number of abortion centers. The correlation between these two variables is .71.

31 A region is defined as the interaction between an economic region (of which there are five in Nepal) and an ecological belt (Terai, hill, or mountain), with 13 regions defined in the DHS.

32 The only outcome for which the conclusions vary when individual DHS surveys are excluded is male sterilization, which significantly increases with an additional abortion center if we exclude the 1996 survey and significantly decreases if we exclude the 2011 survey, whereas it has a statistically insignificant effect if we include all four surveys or exclude the 2001 or 2006 surveys. We therefore conclude from the results in Table 6 that there is no robust evidence of a change in male sterilization, as in the main analysis.

33 We also repeated the analysis excluding the capital Kathmandu, which has the largest number of abortion facilities of all districts. The estimated odds ratios are very similar to those obtained with the whole sample, but estimates become much less precisely estimated due to the loss in variation in our abortion supply variable, and therefore most odds ratios become statistically insignificant. Full results are available on request.

34 The effect estimated here is based on the variation over time in local availability of abortion centers across districts. It may therefore be an underestimate of the true substitution effect because women may be able to travel to abortion centers located outside their district (e.g., in the capital Kathmandu).
