# Supplementary material for: Population Policy: Abortion and Modern Contraception Are Substitutes
Source: Demography. 2016 Jul 6;53(4):979–1009. doi: 10.1007/s13524-016-0492-8 (PMC5016566; doi:10.1007/s13524-016-0492-8)
Supplement: Supplementary file 1 — (DOCX 556 kb) [file 13524_2016_492_MOESM1_ESM.docx]

**Online Resource 1**

**Population Policy: Abortion and Modern Contraception Are Substitutes**

Grant Miller and Christine Valente

Figure S1: Abortion and Contraception Trends in Former Communist Countries with Complete Abortion Data


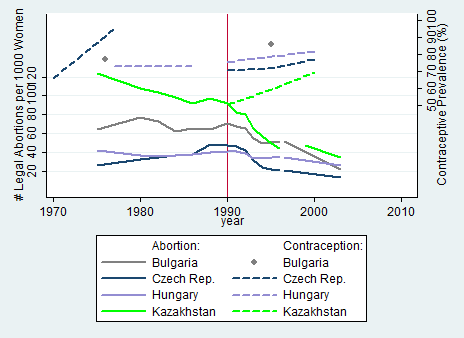


*Sources:* Abortion: 1975–1996 from Henshaw et al. (1999), 1996–2003 from Sedgh et al. (2007). Contraception: 1970–1987 from Mauldin and Segal (1988), 1990–2000 from United Nations (2004). Contraception figures for 1970–1987 labeled “Czech Republic” are aggregate figures for Czechoslovakia.

**Fig. S2** Analytical framework

|  |  |  |  |  |
| --- | --- | --- | --- | --- |
| C=1 | ${1- p}_{f}$ | no pregnancy |  | A=0 |
|  | ${pf}$ |  |  |  |
|  |  | pregnancy |  | A=0 |
|  |  |  |  |  |
| C=0 |  |  |  | A=1 |

Table S1: Results by age group

| Dependent variable: =1 if Modern Method, 0 otherwise | | | | | | | |
| --- | --- | --- | --- | --- | --- | --- | --- |
|  | (1) | (2) | (3) | (4) | (5) | (6) | (7) |
| Age Group: | 15-19 | 20-24 | 25-29 | 30-34 | 35-39 | 40-44 | 45-49 |
|  | | | | | | | |
| Number of Abortion Centers | 0.948*** | 0.971*** | 0.977*** | 0.956*** | 0.974* | 1.000 | 0.994 |
|  | (0.0170) | (0.0076) | (0.0076) | (0.0078) | (0.0146) | (0.0109) | (0.0150) |
|  |  |  |  |  |  |  |  |
| Observations | 2578 | 5868 | 6415 | 5435 | 4678 | 3852 | 2982 |

Output omitted for the following variables: three dummy variables indicating DHS waves, district fixed-effects, and controls for urban location, age at interview, religion, and education summarized in Table 1 Panel C. See also Notes under Table 2. *** p<0.01, ** p<0.05, * p<0.10.

| Table S2: Results Obtained Using Recall Data   \| Dependent Variable: =1 if any method, 0 otherwise \| \| \| \| \| \| \| --- \| --- \| --- \| --- \| --- \| --- \| \|  \|  \| District-Specific Trends \| \| \|  \| \|  \| (1) \| (2) \| (3) \| (4) \| (5) \| \|  \| Baseline \| Linear Trends \| Quadratic Trends \| Cubic Trends \| Placebo Test \| \| Number of Abortion Centers \|  \|  \|  \|  \|  \| \| 0.977*** \| 0.970*** \| 0.969** \| 0.984** \| 0.965** \| \| in the woman’s district \| (0.0045) \| (0.0111) \| (0.0143) \| (0.0082) \| (0.0138) \| \| Number of Abortion Centers 12 months later \|  \|  \|  \|  \| 1.008 \| \|  \|  \|  \|  \|  \| (0.0120) \| \|  \|  \|  \|  \|  \|  \| \| District Dummies \| Yes \| Yes \| Yes \| Yes \| Yes \| \| Covariates \| Yes \| Yes \| Yes \| Yes \| Yes \| \| Observations \| 923886 \| 923886 \| 923886 \| 923886 \| 757050 \|   Sample of married women interviewed in the 2006 and 2011 DHS surveys who were not sterilized and whose husbands were not sterilized prior to March 2004. Period included: April 2000 (start of the calendar period for the 2006 DHS) to February 2010 (last month for which we have data on registration of abortion centers). Output omitted across all columns for the following variables: a dummy variable for DHS 2011, district fixed-effects, month/year dummies (e.g., May 2008), and controls for urban location, age at interview, religion, and education. The sample in Column (5) is smaller as it excludes the last 12 calendar months for which we have data on current abortion centers but not future abortion centers. Source: Authors' calculations using Demographic and Health Surveys of Nepal (1996, 2001) and Technical Committee for Implementation of Comprehensive Abortion Care (2010). *** p<0.01, ** p<0.05, * p<0.10. |
| --- | --- | --- | --- | --- | --- | --- | --- | --- | --- | --- | --- | --- | --- | --- | --- | --- | --- | --- | --- | --- | --- | --- | --- | --- | --- | --- | --- | --- | --- | --- | --- | --- | --- | --- | --- | --- | --- | --- | --- | --- | --- | --- | --- | --- | --- | --- | --- | --- | --- | --- | --- | --- | --- | --- | --- | --- | --- | --- | --- | --- | --- | --- | --- | --- | --- | --- | --- | --- | --- | --- | --- | --- | --- | --- | --- | --- | --- |
